# Calcium/P53/Ninjurin 1 Signaling Mediates Plasma Membrane Rupture of Acinar Cells in Severe Acute Pancreatitis

**DOI:** 10.3390/ijms241411554

**Published:** 2023-07-17

**Authors:** Chehao Lee, Guang Xin, Fan Li, Chengyu Wan, Xiuxian Yu, Lijuan Feng, Ao Wen, Yu Cao, Wen Huang

**Affiliations:** Department of Emergency Medicine and Laboratory of Ethnopharmacology, Tissue-Orientated Property of Chinese Medicine Key Laboratory of Sichuan Province, West China School of Medicine, West China Hospital, Sichuan University, Chengdu 610041, China

**Keywords:** nerve-injury-induced protein 1, plasma membrane rupture, severe acute pancreatitis

## Abstract

Ninjurin 1 (NINJ1) is a double-transmembrane cell-surface protein that might mediate plasma membrane rupture (PMR) and the diffusion of inflammatory factors. PMR is a characteristic of acinar cell injury in severe acute pancreatitis (SAP). However, the involvement of NINJ1 in mediating the PMR of acinar cells in SAP is currently unclear. Our study has shown that NINJ1 is expressed in acinar cells, and the expression is significantly upregulated in sodium-taurocholate-induced SAP. The knockout of NINJ1 delays PMR in acinar cells and alleviates SAP. Moreover, we observed that NINJ1 expression is mediated by Ca^2+^ concentration in acinar cells. Importantly, we found that Ca^2+^ overload drives mitochondrial stress to upregulate the P53/NINJ1 pathway, inducing PMR in acinar cells, and amlodipine, a Ca^2+^ channel inhibitor, can reduce the occurrence of PMR by decreasing the concentration of Ca^2+^. Our results demonstrate the mechanism by which NINJ1 induces PMR in SAP acinar cells and provide a potential new target for treatment of SAP.

## 1. Introduction

Acute pancreatitis (AP) is a digestive system disease associated with pancreatic inflammation and edema [1]. It can be classified into different categories based on severity: mild acute pancreatitis without necrosis or organ failure, moderate pancreatitis with sterile necrosis or transient organ failure, moderately severe acute pancreatitis with infected necrosis or persistent organ failure, and severe acute pancreatitis (SAP) with infected necrosis and persistent organ failure [2,3]. Various factors, such as gallstones, alcoholism, and hyperlipidemia, can contribute to the development of AP [1]. The worldwide annual incidence of acute pancreatitis is 34 cases per 100,000 individuals, with an average annual percentage increase of 3.07% [4,5]. Conventional treatment for pancreatitis usually includes drainage, the administration of antibiotics or the surgical removal of necrotic tissue, dietary control, pain management, or fluid replacement. However, the majority of these treatments are symptomatic, and our understanding of the underlying causes, such as gallstones or alcohol abuse, remains limited [6,7]. Approximately 20% of AP cases may progress to SAP, which is characterized by plasma membrane rupture (PMR), the release of damage-associated molecular patterns (DAMPs), and inflammatory factors. This condition can lead to a high mortality rate, making SAP a significant clinical challenge as it lacks an effective pharmacological treatment option due to the limited understanding of its causes and underlying mechanisms [8,9].

Ninjurin1 (NINJ1) was initially identified as a transmembrane protein that is upregulated in response to nerve injury [10]. NINJ1 has been recognized as a mediator of immune cells by facilitating cell adhesion through homophilic interactions and interaction of the same protein, mediated by its amino acid 26-37 domain [11]. Recent studies have reported the involvement of NINJ1 in PMR [12], which is a critical event in the development of SAP. The recruitment of immune cells and the occurrence of PMR have a significant impact on the progression of SAP. However, the role of NINJ1 in pancreatic acinar cells and its relationship with SAP are still not fully understood.

Ca^2+^ overload is a key event in the development of SAP, which can result in cell necrosis and ultimately lead to PMR [8,13]. The endoplasmic reticulum (ER) functions as an intracellular Ca^2+^ reservoir in acinar cells and plays a crucial role in regulating intracellular Ca^2+^ levels. Stimulation by bile acids can trigger the release of Ca^2+^ from ER into the cytosol through Ca^2+^ inositol 1,4,5-trisphosphate receptor (IP3R) channels, a member of the family of intracellular calcium release channels located in the ER [8]. As a result, a massive amount of stored Ca^2+^ is released from the ER into the cytoplasm. This accumulation activates calcium-release-activated calcium modulator 1 (Orai1), facilitating the entry of extracellular Ca^2+^ into acinar cells, resulting in persistent cytoplasmic Ca^2+^ overload [13]. Such an overload can contribute to the premature activation of trypsin, production of inflammatory cytokines, and mitochondrial stress [14]. Finally, acinar cell PMR could induce cellular necrosis [15]. Ca^2+^ and NINJ1 can eventually contribute to PMR; however, the correlation between Ca^2+^ and NINJ1 is still unclear.

Elevated cytoplasmic Ca^2+^ levels can activate the mitochondrial calcium uniporter (MCU) and induce the transfer of Ca^2+^ into the mitochondria. The primary physiological role of mitochondria in the cell is to synthesize ATP, which provides energy for cellular life activities. The continuous redox reactions and electron transport chain in the inner mitochondrial membrane result in a potential difference between the inner and outer mitochondrial membranes, known as the mitochondrial membrane potential (ΔΨm) [8,13]. The excessive influx of Ca^2+^ can also lead to mitochondrial dysfunction, oxidative stress, and cellular damage [16]. Additionally, excessive ROS could induce DNA damage and contribute to the upregulation of P53 expression [17]. It receives various signals related to cellular health and determines whether to initiate cell division. If the cell is irreparably damaged, P53 triggers apoptosis or necrosis [18,19,20]. Interestingly, activated P53 binds to the promoter region of NINJ1, which leads to the upregulation of NINJ1 expression [21]. Therefore, Ca^2+^ overload can promote mitochondrial stress and induce P53 to regulate NINJ1.

Amlodipine (AML) is a dihydropyridine Ca^2+^ channel blocker, which is commonly used to treat hypertension and angina by relaxing blood vessels and reducing the workload on the heart [22]. It can also prevent heart and blood vessel problems such as heart attacks and strokes. Recent studies have shown that AML may reduce the expression of NINJ1 in endothelial cells [23], but they did not explain how NINJ1 is affected from the perspective of Ca^2+^.

The aim of this study is to investigate the underlying mechanisms of SAP and explore the role of NINJ1 in pancreatic acinar cells and its relationship with Ca^2+^ overload. The study aims to obtain a deeper understanding of the involvement of Ca^2+^ and NINJ1 in the inflammatory process of pancreatitis, providing scientific evidence for the development of novel therapeutic strategies for SAP.

## 2. Results

### 2.1. NINJ1 Is Expressed and Activated in SAP Acinar Cells

**Figure 1:**
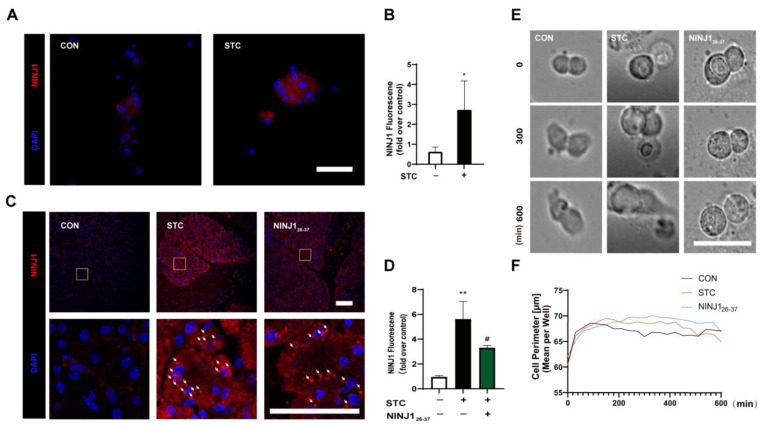
NINJ1 accumulates on STC-SAP mouse acinar cells and affects PMR. (**A**) Primary mouse acinar cell treated with 5 mM STC for 50 min, then stained with IF. IF: NINJ1 observed; scale bar = 100 μm. (**B**) The quantitative plot of NINJ1 fluorescence levels (*n* = 5). (**C**) NINJ1_26-37_ was intraperitoneally injected before modeling (3 and 6 mg/kg), once a day, three times in total; the SAP model was induced by retrograde injection of 3.5% STC into the pancreaticobiliary duct. Samples were taken 24 h later. Yellow square = enlarged area. White arrow = oligomerization. Scale bar = 100 µm. (**D**) The quantitative plot of NINJ1 fluorescence levels. (*n* = 3). (**E**) Mouse pancreatic acinar cells 266-6 were photographed using HCS real-time imaging. Scale bar = 50 µm. (**F**) An HCS data analysis system was used to analyze the changes in the cell perimeter at different time points. SAP: severe acute pancreatitis, STC: sodium taurocholate, PMR: plasma membrane rupture, IF: immunofluorescence, HCS: high-content screening. All data are presented as mean ± SEM; * *p* < 0.05, ** *p* < 0.01 vs. the control group. ^#^ *p* < 0.05 vs. the STC group.

To confirm the expression of NINJ1 in acinar cells, primary mouse acinar cells were extracted and incubated with sodium taurocholate (STC) for 50 min to establish an in vitro model of SAP. Immunofluorescence staining was performed to detect the expression of NINJ1; the results demonstrated that NINJ1 was expressed in acinar cells, and its expression was significantly upregulated in the STC group (Figure 1A,B). This finding that NINJ1 is expressed in acinar cells suggests that it may play a role in the pathogenesis of SAP.

To investigate the function of NINJ1, NINJ1_26-37_ (a blocking peptide) is utilized to block the NINJ1-adhesive fragment sequence of NINJ1 [24], which has been reported to inhibit platelet PMR [25]. A primary acinar cell necrosis experiment was conducted to examine the effect of NINJ1_26-37_ on STC-SAP acinar cells. The results demonstrated that the STC group induced acinar cell necrosis, while the NINJ1_26-37_ (10 μM) group markedly reduced the necrosis rate of acinar cells (Supporting Information, Figure S1A,B).

To evaluate the potential therapeutic effect of NINJ1_26-37_ on SAP in vivo, a classic SAP model was established by administering STC. Histological scoring demonstrated a significant increase in tissue, necrosis, inflammation, and edema in the SAP group, along with elevated amylase and lipase serum levels (Supporting Information, Figure S1C–E). Subsequent treatment with NINJ1_26-37_ significantly attenuated tissue necrosis and inflammatory infiltration and resulted in decreased levels of serum amylase and lactate dehydrogenase (LDH) compared to the STC group (Supporting Information, Figure S1E).

Additionally, immunofluorescence staining was performed on mouse pancreas samples to investigate the precise localization of NINJ1 expression and the impact of NINJ1_26-37_ interventions. The results revealed the expression of NINJ1 on the acinar cell membrane, which was significantly upregulated in the STC group. Treatment with NINJ1_26-37_ suppressed NINJ1 expression and oligomerization in SAP pancreatic acinar cells (Figure 1C,D).

Taken together, our in vitro and in vivo experiments collectively demonstrate that NINJ1 is expressed on the acinar cell membrane and its expression is significantly upregulated in STC-SAP acinar cells. Moreover, the inhibition of NINJ1 expression by NINJ1_26-37_ could alleviate SAP, suggesting that targeting NINJ1 could be a promising therapeutic strategy for this disease.

The 266-6 mouse pancreatic cell lines were employed, and the suitable concentrations for STC-SAP modeling (1.5 mM) and NINJ1_26-37_ administration (35 μM) were determined through CCK-8 assays (Figure S2). Based on the aforementioned concentration, we conducted real-time imaging for 600 min using high-content screening (HCS) to observe cell morphology. We found that the 266-6 acinar cells in the control group did not rupture and produce new cells (Supporting Video S1). In the STC group, the acinar cells began to rupture at around 600 min. (Supporting Video S2). In the NINJ1_26-37_ group, the acinar cells did not undergo rupture within 600 min (Figure 1E, Supporting Video S3). An analysis of the cell perimeters showed no statistical difference between the control group, the STC group, and the NINJ1_26-37_ group in the 0–300 min time period (Figure 1F, Supporting Information Figure S3A). In the 300–600 min time period, the cell perimeter of the STC group was significantly larger compared to the control group (Figure 1F, Supporting Information Figure S3B). The cell perimeter in the NINJ1_26-37_ group exhibited a significant increase compared to the STC group.

### 2.2. NINJ1 Knockout Inhibits PMR of Acinar Cells and Ameliorated SAP

**Figure 2:**
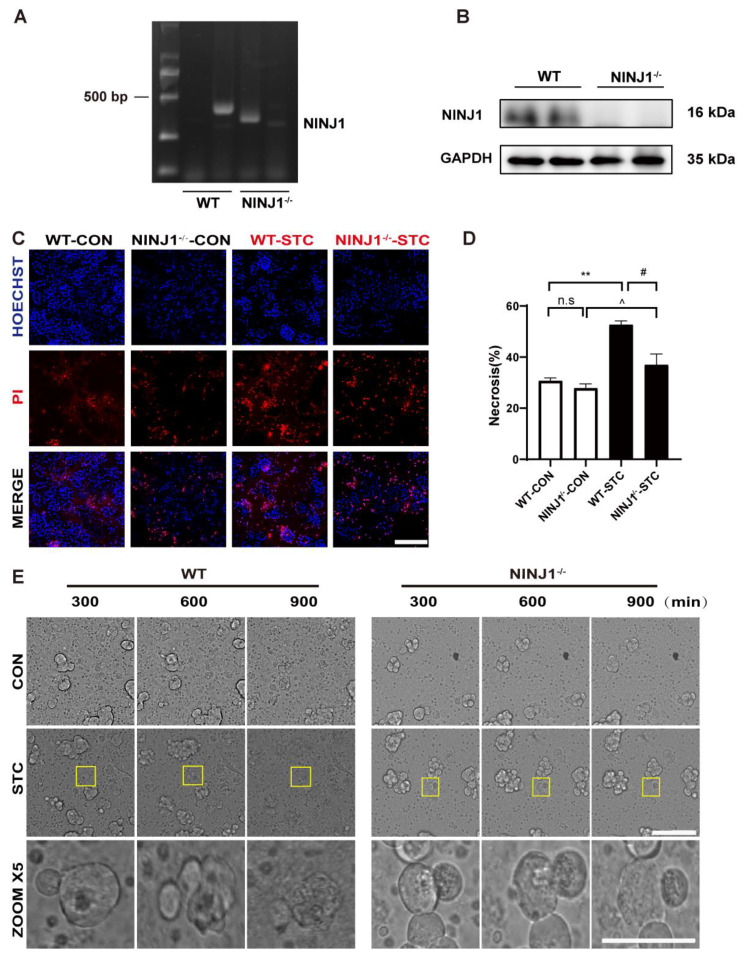
Protective effect of NINJ1^−/−^ on acinar cells. (**A**) NINJ1 DNA expression in acinar cells from NINJ1^−/−^ mice. (**B**) Western blot detected NINJ1 protein expression in the NINJ1^−/−^ mice acinar cells. GAPDH was used as the loading control (*n* = 3). (**C**) Representative fluorescence plot of PI/Hoechst 33,342 staining in primary mouse acinar cells. Scale bar = 200 µm. (**D**) PI/Hoechst 33,342 staining necrosis bar chart of primary mouse acinar cells (*n* = 3). Data are presented as mean ± SEM, ** *p* < 0.01 indicates WT-CON vs. WT-STC, ^#^ *p* < 0.05 indicates WT-STC vs. KO-STC; ^^^ *p* < 0.05 indicates WT-STC vs. NINJ1^−/−^-STC. n.s = no significance. (**E**) HCS real-time imaging of mouse pancreatic acinar cell and representative pictures were selected from the periods of 300, 600, and 900 min in WT-CON group, WT-STC group, NINJ1^−/−^-CON group, NINJ1^−/−^-STC group (*n* = 3). STC: sodium taurocholate. HCS: high-content screening. Scale bar = 50 µm.

To further confirm the role of NINJ1 in SAP, NINJ1^−/−^ mice were generated (Figure 2A,B). Primary acinar cells from WT and NINJ1^−/−^ mice were co-incubated with the STC separately in an experiment to induce acinar cells. There was no significant difference in necrosis rate between the NINJ1^−/−^-CON group and the WT-CON group. Furthermore, following treatment with STC, the NINJ1^−/−^-STC group exhibited a significantly lower necrosis rate compared to the WT-STC group (Figure 2C,D), suggesting that NINJ1^−/−^ acinar cells have a protective effect against STC-induced cell death.

We conducted an HCS real-time imaging of the plasma membrane in primary acinar cells from NINJ1^−/−^ mice over a 900 min duration. The NINJ1^−/−^ group exhibited a significant decrease in PMR under STC treatment compared to the WT group (Figure 2E, Supporting Videos S4–S7). Concurrently, we observed a significant increase in cell perimeter in the NINJ1^−/−^ group compared to the WT group and in the NINJ1^−/−^-STC group compared to the NINJ1^−/−^-CON group during the 300–600 and 600–900 min intervals (Figure S4A–D). These findings suggest that NINJ1^−/−^ can effectively delay the onset of PMR. Concurrently, we observed a significant increase in cell perimeter in the NINJ1^−/−^ group compared to the WT group and in the NINJ1^−/−^-STC group compared to the NINJ1^−/−^-CON group during the 300–600 and 600–900 min intervals (Figure S4A–D). These findings suggest that NINJ1^−/−^ can effectively delay the onset of PMR.

### 2.3. NINJ1 Expression Is Mediated by Ca^2+^

**Figure 3:**
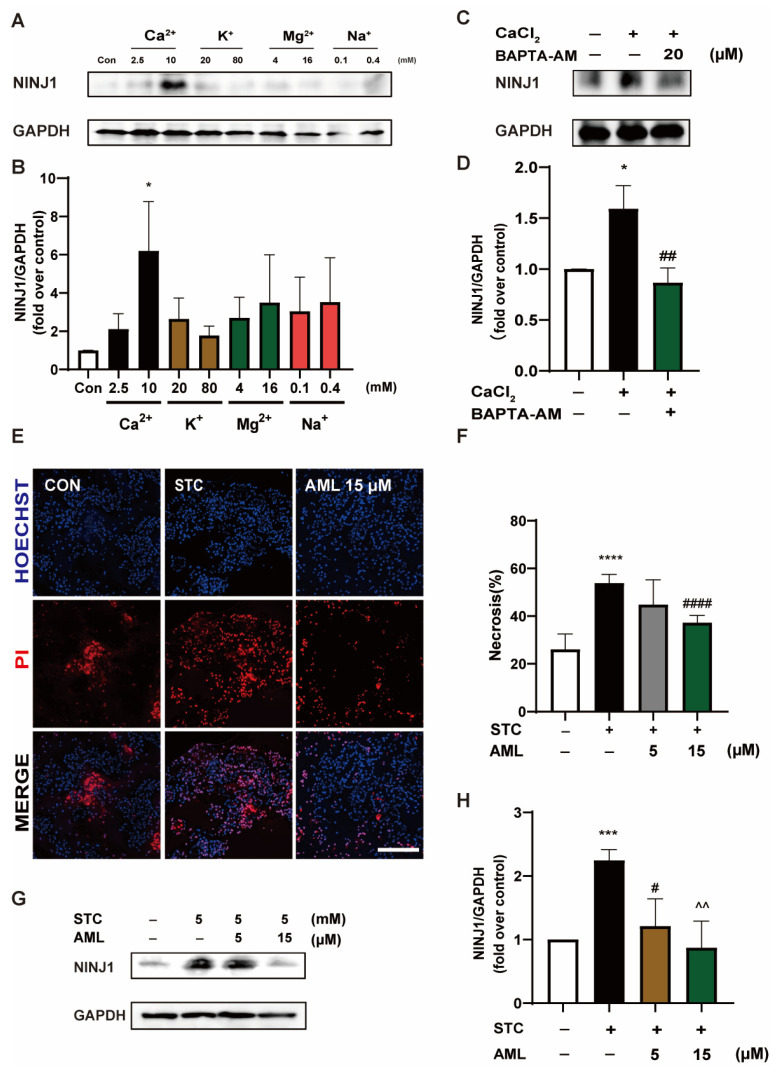
NINJ1 expression is mediated by Ca^2+^ concentration. (**A**) Primary mouse acinar cells were incubated with different metal ions for 50 min, and the proteins were extracted for Western blot experiments to detect the effects of Ca^2+^, K^+^, Mg^2+^, and Na^+^ on NINJ1. (**B**) The quantitative plot of NINJ1 protein levels (*n* = 3). (**C**) Ca^2+^ (10 mM) was added with mouse primary acinar cells and incubated with BAPTA-AM for 50 min, and the protein was extracted for Western blot analysis to detect the effect of BAPTA-AM on NINJ1. (**D**) The quantitative map of NINJ1 protein level (*n* = 3). (**E**) Primary mouse acinar cells were incubated with AML (5 and 15 μM) and incubated with STC (5 mM) for 50 min. Representative fluorescence plot of PI/Hoechst 33342 staining in primary mouse acinar cells. Scale bar = 200 µm. (**F**) Column of PI/Hoechst 33342 staining necrosis in primary mouse acinar cells (*n* = 5). (**G**) Primary mouse acinar cells were incubated with AML (15 μM) combined with STC (5 mM) for 50 min, and the proteins were extracted for Western blot experiments. (**H**) The quantitative lot of NINJ1 protein levels (*n* = 3). STC: sodium taurocholate; AML: amlodipine. All data are presented as mean ± SEM, * *p* < 0.05, *** *p* < 0.001, **** *p* < 0.0001 vs. the control group. ^#^ *p* < 0.05, ^##^ *p* < 0.01, ^####^ *p* < 0.0001, ^^^^ *p* < 0.01 vs. the STC group.

Metal ions play a critical role in cells’ physiological and pathological processes, and their dysfunction may ultimately contribute to PMR [26,27]. To investigate the association between metal ions and NINJ1 in acinar cells, we conducted experiments where primary mouse acinar cells were incubated with calcium, potassium, magnesium, and sodium. Subsequently, proteins extracted via Western blot analysis revealed a notable upregulation of NINJ1 expression in cells incubated with Ca^2+^ (Figure 3A,B). To further validate the influence of Ca^2+^ on NINJ1, we employed the Ca^2+^ chelator BAPTA-AM to modulate NINJ1 expression. As anticipated, the presence of BAPTA-AM led to a substantial reduction in NINJ1 protein expression (Figure 3C,D). Nevertheless, BAPTA-AM has limited clinical applicability due to its inadequate targeting, prompting us to investigate a Ca^2+^ channel antagonist, AML, commonly employed for hypertension [28]. Previous studies have reported that AML can inhibit NINJ1 expression in human endothelial cells [23]. Given the crucial role of Ca^2+^ in the pathological mechanism of SAP, we hypothesized that AML may be effective against SAP.

To examine this hypothesis, we performed in vitro experiments and discovered that AML at a concentration of 15 μM effectively suppressed acinar cell necrosis (Figure 3E,F) and attenuated NINJ1 expression (Figure 3G,H). These findings indicate that the expression of NINJ1 is regulated by the Ca^2+^ level. Furthermore, AML demonstrated an effective inhibition of NINJ1 expression and a reduction in acinar cell necrosis, thereby suggesting the potential therapeutic utility of AML in the treatment of SAP.

### 2.4. NINJ1 Inhibition by AML Improves SAP in Mice

**Figure 4:**
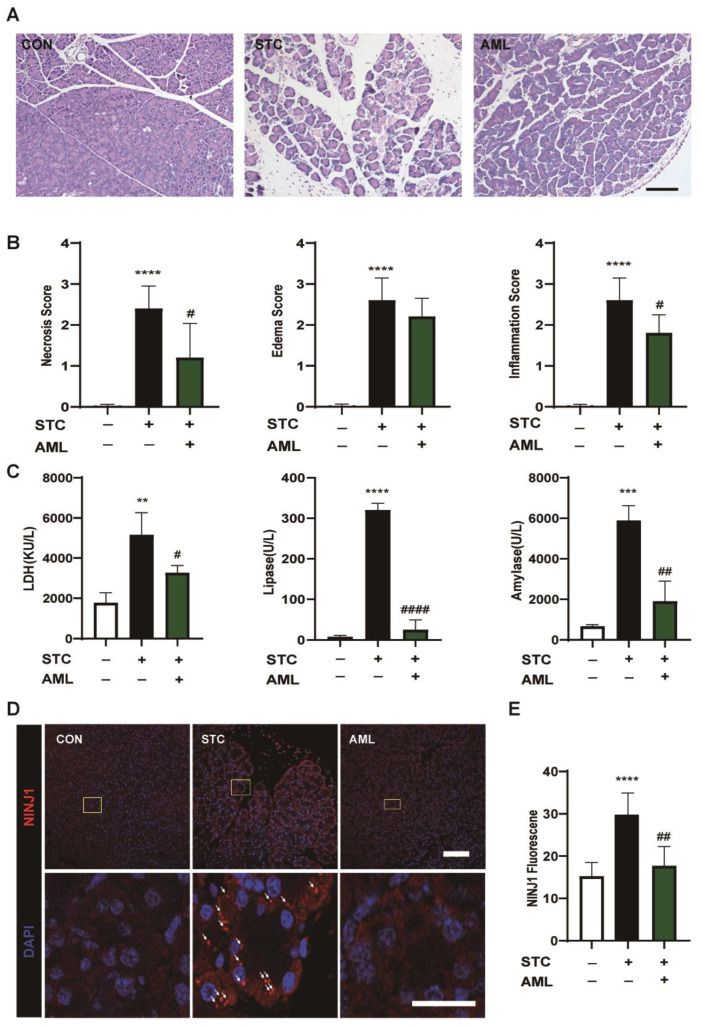
Effect of AML on pancreatic tissue in STC-SAP mice. (**A**) AML (6 mg/kg) was intraperitoneal injected once a day three times before modeling; SAP was induced by retrograde injection of 3.5% STC into the pancreaticobiliary duct. Samples were taken 24 h later. Scale bar = 50 µm. (**B**) The histopathological score of mouse pancreas (*n* = 5). (**C**) Serum levels of lactate dehydrogenase, lipase, and amylase were measured (*n* = 3). (**D**) Immunofluorescence plot of NINJ1 on pancreatic tissue, Yellow square = enlarged area. White arrow = oligomerization. Scale bar = 100 µm. (**E**) The quantitative map of NINJ1 fluorescence levels (*n* = 5). STC: sodium taurocholate. SAP: severe acute pancreatitis. AML: amlodipine. All data are presented as mean ± SEM, ** *p* < 0.01, *** p < 0.001, **** p < 0.0001 vs. the control group, ^#^ *p* < 0.05, ^##^ *p* < 0.01, ^####^ *p* < 0.0001 vs. the STC group.

To further validate the effectiveness of AML in SAP, we administered AML (6 mg/kg) to mice with STC-induced SAP. An evaluation of histological scores revealed that AML attenuated pancreatic edema, inflammation, and necrosis in pancreatic tissue (Figure 4A,B). Additionally, the AML group exhibited significant reductions in LDH, lipase, and amylase levels (Figure 4C). Furthermore, the immunofluorescence assay provided additional evidence of the significant inhibitory effect of AML on NINJ1 expression (Figure 4D,E). These results demonstrate the substantial protective effects of AML against SAP and its significant inhibitory effect on NINJ1 expression.

### 2.5. Inhibition of Ca^2+^Channels Protects ΔΨm and Decreases P53 Expression

**Figure 5:**
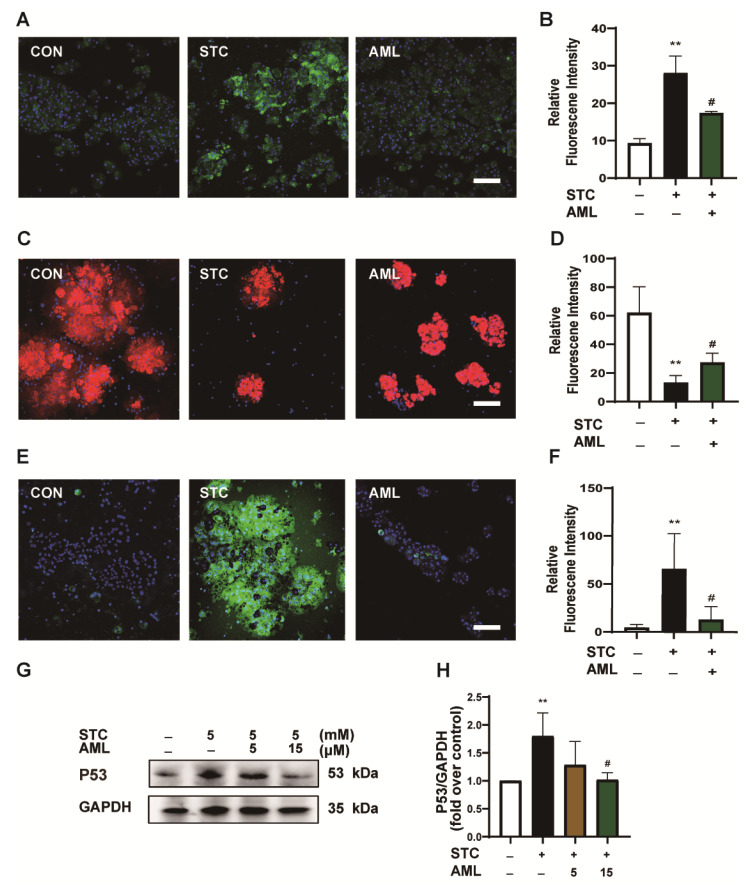
AML via inhibition of intracellular Ca^2+^ to inhibit ROS-P53. (**A**) Mouse primary acinar cells incubated with 15 μM AML for 30 min; Fluo-4 AM was added for another 20 min; STC (5 mM) was added before microscopy, and then observed under confocal microscope. A representative Fluo-4 AM staining. Scale bar = 100 μm. (**B**) The quantitative plot of Fluo-4AM fluorescence levels (*n* = 3). (**C**) Primary mouse acinar cells were incubated with 15 μM AML for 30 min, followed by the addition of TMRM for another 20 min, and then observed under confocal microscope. The representative TMRM staining. Scale = 100 μm. (**D**) A quantitative map of TMRM fluorescence levels (*n* = 3). (**E**) Primary mouse acinar cells were incubated with 15 μM AML for 30 min; then, DCFH-DA was added and co-incubated for another 20 min before observation under confocal microscope. The representative DCFH-DA staining. Scale = 100 μm. (**F**) The quantitative plot of DCFH-DA fluorescence levels (*n* = 3). (**G**) The primary mouse acinar cells were incubated with AML and STC for 50 min, and the protein was extracted for Western blot analysis to detect the effect of AML on P53. (**H**) The quantitative plot of P53 protein levels (*n* = 4). AML: amlodipine, STC: sodium taurocholate. All data are presented as mean ± SEM, ** *p* < 0.01 vs. the control group; ^#^ *p* < 0.05 vs. the STC group.

To investigate the impact of AML on the Ca^2+^levels, we initially utilized Fluo-4 AM, a Ca^2+^ probe, to assess the cytoplasmic Ca^2+^ levels. The STC group exhibited a significant increase in Ca^2+^ levels, while the AML group showed a decrease in intracellular Ca^2+^ concentration (Figure 5A,B). Furthermore, we employed TMRM, a probe for measuring ΔΨm, to assess changes in ΔΨm. The STC group exhibited a reduction in ΔΨm compared to the control group, whereas the AML group showed higher ΔΨm levels than the STC group (Figure 5C,D). Moreover, DCFH-DA, a probe for ROS, was utilized to monitor the ROS changes in each group. The level of ROS was significantly higher in the STC group compared with the control group, while it was significantly lower in the AML group (Figure 5E,F). Additionally, we measured ROS indicators superoxide dismutase (SOD) and malondialdehyde (MDA). The experimental results showed decreased levels of SOD and increased levels of MDA in the STC group. AML increased SOD and reduced MDA levels in SAP acinar cells (Supporting Information, Figure S3). Furthermore, we observed that the expression of P53 was upregulated in the STC group and downregulated after treatment with AML (Figure 5G,H). Collectively, STC can induce Ca^2+^ overload in acinar cells. This overload induces the loss of ΔΨm, which disrupts the mitochondrial respiratory chain, promotes ROS overproduction, and upregulates P53 expression. AML decreases cytosolic Ca^2+^ levels, thereby mitigating mitochondrial damage and downregulating P53 expression.

### 2.6. P53 Upregulates NINJ1 to Induce PMR

**Figure 6:**
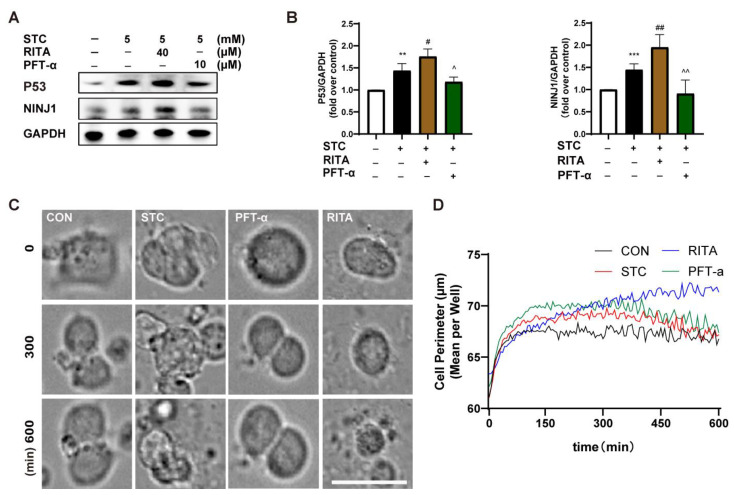
NINJ1 is regulated by P53 and affects PMR. (**A**) Primary mouse acinar cells were incubated with STC, RITA, and PFT-α for 50 min. The effects of STC on P53 and NINJ1 were detected by Western blot. (**B**) The quantitative plot of P53 (*n* = 4) and NINJ1 (*n* = 5) protein levels. (**C**) 266-6 mouse pancreatic acinar cells were captured by HCS real-time imaging. Scale bar = 50 µm (**D**) An HCS data analysis system was used to analyze the changes in cell perimeter at different time points. STC: sodium taurocholate; RITA: P53 agonist; PFT-α: P53 inhibitor. HCS: high-content screening. PMR: plasma membrane rupture. All data are presented as mean ± SEM, ** *p* < 0.01, *** *p* < 0.001 vs. the control group, ^#^ *p* < 0.05, ^##^ *p* < 0.05, ^^^ *p* < 0.05, ^^^^ *p* < 0.05 vs. the STC group.

P53, as a transcriptional factor, binds to the promoter of NINJ1 to enhance its expression in MCF7 cells, and NINJ1 could promote PMR [11,21]. To further confirm the involvement of P53 in regulating NINJ1 expression in acinar cells, we incubated primary acinar cells with either P53 agonist RITA or inhibitor PFT-α. We conducted a necrosis examination on acinar cells to identify effective concentrations. Treatment with RITA at 10 µM significantly promoted acinar cell necrosis, whereas PFT-α at 40 μM protected against acinar cell necrosis (Supporting Information, Figure S6A,B). Subsequently, the expression of P53 and NINJ1 was increased in acinar cells treated with RITA and decreased in those treated with PFT-α (Figure 6A,B). These results indicate that P53 can regulate the expression of NINJ1 in primary acinar cells and may play a crucial role in the pathogenesis of PMR.

To further demonstrate the regulatory role of P53 in mediating PMR via NINJ1, a CCK-8 assay was used to screen appropriate RITA (3 μM) and PFT-α (10 μM) concentrations for 266-6 (Supporting Information, Figure S2C,D). According to HCS real-time imaging for 600 min, the cell perimeter of the STC group was significantly larger than that of the control group. The perimeter of the PFT-α group increased considerably at 100 min and started to decrease at 300 min, and it eventually returned to the level of the control group at 600 min. The cell perimeter of the RITA group was smaller than that of the STC group for the first 300 min, and it increased significantly between 300 and 600 min, exceeding 70 μm at 600 min (Figure 6C,D, Supporting Information Figure S7A,B, Supporting Video S8–S11). These results suggest that inhibiting NINJ1 could promote the maintenance of a larger cell membrane perimeter to prevent rupture. When P53 was upregulated, the cell perimeter was smaller than the STC group during the first 300 min and directly increased to 70 μm during the last 300 min. It is possible that NINJ1 activation caused cell membrane rupture, leading to the induction of DAMPs, extensive cell rupture, and an increased cell perimeter. In conclusion, P53 plays a role in regulating NINJ1 expression and mediating PMR in SAP acinar cells.

## 3. Discussion

SAP is an inflammatory disease of the pancreas characterized by tissue damage, PMR, and the necrosis of acinar cells. It is also referred to as necrotizing pancreatitis due to its features [29]. Currently, there is no effective pharmacological treatment to halt the progression of the disease. Therefore, it is necessary to identify new protein targets.

This article aimed to validate the expression and function of NINJ1 in pancreatic acinar cells. Initially, we observed the upregulation of NINJ1 in primary acinar cells upon STC stimulation. To investigate the impact of NINJ1 on acinar cells, we employed NINJ1_26-37_, which primarily diminishes immune cell recruitment by targeting the adhesive domain of NINJ1 [24]. Previously, our group study demonstrated that NINJ1_26-37_ diminishes platelet PMR, thereby suppressing adhesion and aggregation [25]. To verify the involvement of NINJ1 in SAP, we conducted in vitro and in vivo experiments, demonstrating the therapeutic effect of NINJ1_26-37_ on SAP. However, whether NINJ1 regulates PMR in acinar cells remains unknown. Consequently, we employed HCS real-time imaging to analyze morphological alterations. Our findings showed that the cell perimeter of 266-6 co-treated with STC and NINJ1_26-37_ was larger than that of the STC group. This indicates that NINJ1_26-37_ could promote the perimeter of acinar cells to reduce PMR by inhibiting NINJ1. The aforementioned experiments provided evidence that NINJ1 inhibition can reduce PMR and alleviate SAP both in vivo and in vitro.

In order to further ascertain the role of NINJ1, we generated NINJ1^−/−^ mice and conducted in vitro experiments to validate its impact on SAP. HCS real-time imaging demonstrated increased cell perimeters compared to the WT group. Moreover, STC treatment led to a significantly prolonged PMR duration in primary acinar cells from NINJ1^−/−^ mice. Based on the findings from necrosis experiments, we can infer that NINJ1 depletion may delay the onset of PMR and mitigate SAP.

We observed a notable upregulation of NINJ1 expression in STC-SAP acinar cells. Current research indicates that STC triggers a pathological elevation of intracellular Ca^2+^, inducing acinar cell PMR. Thus, we hypothesize that NINJ1 expression might be related to the changes in intracellular Ca^2+^ levels. To validate this hypothesis, we co-cultured acinar cells with various metal ions and observed a significant elevation in NINJ1 expression upon Ca^2+^ treatment, which subsequently decreased following BAPTA-AM treatment.

AML, as a dihydropyridine Ca^2+^ channel blocker, has the potential to decrease the expression of NINJ1 through the inhibition of ROS and NF-κB induced by ER stress [23]. However, the precise mechanism underlying AML’s inhibition of ROS and ER stress remains unexplored [23]. Our experiments demonstrated that AML provided relief from SAP both in vivo and in vitro. Furthermore, we observed that AML reduced cytoplasmic Ca^2+^ levels, which protected against mitochondrial dysfunction and the loss of ΔΨm. This led to a decrease in ROS production and facilitated the expected degradation of P53, preventing acinar cell death. Nevertheless, AML is known to be a specific inhibitor of L-type voltage-gated Ca^2+^ channels, which are clearly absent from pancreatic acinar cells [13]; therefore, the specific regulatory mechanism of AML in SAP will be the focus of our future research.

P53 elicits cellular repair or death, both of which can be attributed to mitochondrial dysfunction and oxidative stress [30,31]. Apoptosis is widely acknowledged as the principal outcome associated with P53 activity. Intriguingly, emerging evidence has gradually suggested that P53 might induce necrosis in acinar cells affected by SAP [19,20], thus bestowing a novel perspective on P53. Apart from NINJ1, other mechanisms may also contribute to acinar cell necrosis. Our findings revealed that RITA (a P53 agonist) diminished the rate of acinar cell survival, whereas PFT-α, a P53 inhibitor, resulted in an elevated survival rate in primary acinar cells and 266-6 cells. Lastly, HCS real-time imaging substantiated the inducement of PMR by RITA and the preventive effect of PFT-α against PMR. Consequently, these findings elucidate the involvement of P53 in mediating PMR through NINJ1 during SAP.

This study, for the first time, elucidated the expression of NINJ1 in pancreatic acinar cells and investigated its role in SAP. Furthermore, we discovered that the expression of NINJ1 is regulated by Ca^2+^ and demonstrated that Ca^2+^ is responsible for the upregulation of P53 through the induction of mitochondrial stress in acinar cells. Lastly, we delved deeper into the mechanism by which P53 regulates NINJ1 and identified its impact on the occurrence of PMR.

## 4. Materials and Methods

### 4.1. Animals

The animal care and experimental procedures used in this article have been approved by the Ethics Committee of West China Hospital, Sichuan University (Chengdu, China), with a date of issue from March 2021 to March 2023, approval NO: 2021929A, and follow the Guide of Laboratory Animal Care and Use (Institute of Laboratory Animal Resources, 1996).

A total of 68 WT and 5 NINJ1^−/−^ male C57BL/6 mice aged 6–10 weeks from GemPharmatech (Shanghai, China) were used in this study. Approximately 4–5 mice were allocated to each cage and raised in the SPF level animal room of West China Hospital, Sichuan University (Chengdu, China). The temperature was maintained at 23–26 °C and the relative humidity was maintained at around 50%. The mice were able to freely obtain specialized feed and sterilized drinking water.

### 4.2. Materials

Sodium taurocholate and Collagenase IV were purchased from Sigma-Aldrich (St. Louis, MO, USA). Hoechst 33342 and propidium iodide were purchased from Yeasen (Shanghai, China). NINJ1_26-37_ (sequence: PPRWGLRNRPIN) and Scramble peptide (sequence: PPRAGLRNRPIN) were purchased from Bootai (Shanghai, China). RITA was purchased from APExBIO (Houston, TX, USA). PFT-α and tetramethylrhodamine methyl ester (TMRM) were purchased from MCE (Monmouth Junction, NJ, USA). Amlodipine (AML) and BAPTA-AM were purchased by Aladdin (Shanghai, China). Protease inhibitor, RIPA, DCFH-DA, Fluo-4 AM streptomycin, and penicillin were purchased from Beyotime (Shanghai, China). Superoxide dismutase (SOD) and malondialdehyde (MDA) were purchased from Solarbio (Beijing, China). Bicinchoninic acid (BCA) protein assay kit, Cell Counting Kit-8 (CCK8), and RIPA lysis buffer were purchased from Meilunbio (Dalian, China). Dulbecco’s Modified Eagle Medium (DMEM) and fetal bovine serum were purchased from GIBCO (Grand Island, NY, USA). All other chemicals were purchased from Sigma-Aldrich (St. Louis, MO, USA) of Merck. LDH assay kits were purchased from Jiancheng (Nanjing, China). Anti-NINJ1 and secondary antibodies were purchased from Bioss (Beijing, China). NINJ1 and MCU antibodies were purchased from Abclonal (Wuhan, China); IP3R antibody was obtained from Santa Cruz Biotechnology (Santa Cru, CA, USA); P53 antibody was purchased from Abcam (Cambridge, UK).

### 4.3. Animal Model of SAP and Treatments

Twenty wild-type C57BL/6 mice were randomly divided into four groups, including a control group, STC group, STC + NINJ1_26-37_ (3 mg/kg) group, and STC + NINJ1_26-37_ (6 mg/kg) group [32]. The other twenty wild-type C57BL/6 mice were randomly divided into four groups, including a control group, STC group, STC + AML (3 mg/kg) group, and STC + AML (6 mg/kg) group [33,34].

NINJ1_26-37_ or AML was dissolved in physiological saline to prepare a 2 mg/mL NINJ1_26-37_ or AML solution. The solution was solubilized under ultrasound and used immediately after preparation. Two days before and after retrograde injection of the pancreatobiliary duct, a daily dose of 3 mg/kg (50 μL) and 6 mg/kg (100 μL) of NINJ1_26-37_ or AML was administrated to different groups through intraperitoneal injection. For the control group and the STC group, the same volume of physiological saline solution was applied (100 μL).

The previously described method was used [35]; the mice were fasted for 12 h but had access to water before the surgery. A 3.5% STC physiological saline solution was prepared and warmed to 37 °C before use. Then, the mice were weighed and labeled. An intraperitoneal injection of tribromoethanol (2.5%, 100–150 μL per mouse) was administered before waiting for the mice to gradually lose consciousness, ensuring no response when pressing their paws.

Using a shaving razor, the abdominal fur was removed from the mice. The mice were secured on a sterile surgical table. Iodine solution was applied to the surgical site for disinfection. Using fine surgical instruments, a midline incision was made through the skin and muscle layers of the abdomen. The duodenum was located and the pancreatic ducts, bile ducts, and duodenal papilla were exposed. Throughout the procedure, moisture was maintained in the abdominal cavity by adding the necessary amount of physiological saline.

The path of the bile duct was traced upwards from beneath the liver, and an arterial clamp was used to secure it. The duodenum was flattened and the syringe was inserted through it, entering the pancreatic duct via the duodenal papilla. The solution was slowly injected into the pancreas using a microinfusion pump at a rate of 5 µL/min. The injection volume was 0.1 mL/100 g for each mouse. In the control group, the same volume of physiological saline solution was injected. After completing the injection, the clamp was removed and the muscle and skin layers were sutured. The area with was disinfected with iodine solution and the mice were positioned on a heating pad to maintain body temperature while monitoring their physiological condition.

Prior to sampling, after 24 h of modeling, the mice were anesthetized using an appropriate volume of tribromoethanol. The mice were secured on a sterile operating table and an upper abdominal incision was made to fully expose the thoracic cavity and heart. The needle tip of the syringe was inserted into the mouse’s cardiac apex for blood extraction. The collected blood was transferred into a sterile 1.5 mL EP tube and left to stand at room temperature for 30 min prior to centrifugation. This was used for the subsequent detection of lipase and amylase. The pancreatic tissue was collected for H&E staining and IF.

### 4.4. Histopathology

The pathological changes in pancreatic tissue were analyzed. Fresh pancreases were soaked in 4% paraformaldehyde for 48 h. Pancreas tissue was embedded in paraffin and cut into 3 μm pieces. Each slice was colored with H&E. The sample was observed under a microscope and the pancreas pathological changes at 200× magnification were evaluated. The concealed evaluation of pancreatic histopathological scores was performed by two pathologists: the score range for necrosis, inflammatory cell infiltration, and edema were all in the range 0–3.

### 4.5. Immunofluorescence Staining

Pancreatic paraffin sections (3 μm) were prepared and blocked with blank goat serum in PBST to 5%, followed by overnight incubation at 4 °C with anti-NINJ1 antibodies (1:100 dilution). The slices were then incubated with secondary antibodies (1 h, 37 °C). After cleaning with PBS, DAPI was used for nuclear staining (10 min, 37 °C) at a dilution of 1:2000. The stained sample was observed with a confocal microscope (Nikon A1plus, Tokyo, Japan) and measured at excitation and emission wavelengths of 640 nm and 700 nm. Finally, the fluorescence area was quantified using ImageJ (version 1.51k).

The isolated primary acinar cells were first exposed to STC for 50 min. They were soaked with 4% paraformaldehyde for 1 h, followed by incubation with anti-NINJ1 antibodies (dilution of 1:100) overnight at 4 °C. Subsequently, the cells were incubated with the corresponding secondary antibodies in the dark for 1 h at 37 °C. Finally, DAPI was used to counterstain the cells for 10 min. The stained specimens were visualized with a confocal microscope (Nikon A1plus, Tokyo, Japan) and measured at excitation and emission wavelengths of 640 nm and 700 nm. Finally, the fluorescence area was quantified using ImageJ (version 1.51k).

### 4.6. Preparation of Pancreatic Acinar Cells

The primary acinar cells used in the experiment were isolated from male C57BL/6 mice [36]. The pancreases of the mice were treated with collagenase IV (200 U/mL) at 37 °C for 20 min. The resulting cells were dissociated mechanically, filtered through a 100 μm cell filter, and centrifuged at 700 rpm for 2 min to collect the cell precipitates. The cells were then diluted by Hepes solution (140 mM NaCl, 4.7 mM KCl, 1.13 mM MgCl_2_, 1 mM CaCl_2_, 10 mM D-glucose, and 10 mM Hepes, adjusted to pH 7.35 with NaOH) and kept at 37 °C.

### 4.7. STC-Induced Acinar Cell Death Measurement In Vitro

The previously described method was used [37]. Primary acinar cells were treated with STC (5 mM) and incubated at 37 °C for 50 min with or without AML (5 and 15 μM), NINJ1_26-37_ (5 and 10 μM), and Scramble peptide (5 and 10 μM). The cells were then stained with propidium iodide (PI: 1 μmol/mL) and Hoechst 33,342 (50 μg/mL) to label the total number of nuclear and necrotic cells characterized by PMR. Images were captured using an Automatic ZEISS AX10 imager A2/AX10 cam HRC (Oberkochen, Germany). The total number of acinar cells demonstrating PI uptake was recorded for each condition to determine the percentage of necrosis, with five independent isolates used for each condition.

### 4.8. Detection of ROS

The previously described method was used [38]; ROS levels in the acinar cells were measured using DCFH-DA (10 μM). DCFH-DA was added to primary acinar cells at 37 °C in the black container for 20 min before cells were washed twice with Hepes solution and subsequently treated with STC and AML for 50 min. The treated cells were placed on the glass slides, and the images were rapidly observed using a confocal microscope (Nikon A1plus, Tokyo, Japan) with excitation at 488 nm and emission at 525 nm. The fluorescence intensity was then analyzed using Image J (version 1.51k).

### 4.9. ΔΨm Measurement

TMRM (1 μM) was used to evaluate ΔΨm. The acinar cells were incubated with STC and AML for a specific duration before TMRM was added and cells were incubated in the dark at 37 °C for 20 min. After loading, the acinar cells were washed with Hepes solution twice and placed under a confocal microscope (Nikon A1plus, Tokyo, Japan) with excitation at 561 nm and emission at 595 nm. The fluorescence intensity was quantified using Image J (version 1.51k).

### 4.10. Detection of Intracellular Calcium Content

To measure Ca^2+^ content in the cytoplasm, the Fluo-4 AM probe was used. The isolated primary acinar cells diluted in Hepes solution without Ca^2+^ were co-incubated with AML (10 μM) and Fluo-4 AM stain for 30 min. After staining, the cells were suspended twice and rapidly observed under a confocal microscope (Nikon A1plus, Tokyo, Japan) with the addition of STC (5 mM). The fluorescence was pictured with excitation at 488 nm and emission at 525 nm. The fluorescence intensity was quantified using Image J (version 1.51k).

### 4.11. Detection of Serum Amylase and Lipase

Blood samples were centrifuged at 3000 rpm for 10 min, and 40 μL blood serum was diluted to 200 μL with pure water. The serum lipase and amylase were measured by an automatic biochemical analyzer (Roche, Mannheim, Germany).

### 4.12. Western Blot Analysis

As previously mentioned [37], protein lysates from acinar cells were prepared using RIPA buffer (RIPA:PMSF = 100:1). A total of 20 μg of protein lysate samples was loaded onto a polyacrylamide gel and separated by electrophoresis. The separated proteins were transferred from the gel to a PVDF membrane using a wet transfer method. Subsequently, the PVDF membrane was blocked with a blocking solution containing milk to prevent the non-specific binding of antibodies. The membrane was incubated with primary antibodies (1:1000) specific to the target proteins, followed by incubation with secondary antibodies (1:10,000) conjugated to enzymes after washing and containing 1% Tween. The protein bands were visualized using chemiluminescent or chromogenic substrates that reacted with the enzymes conjugated to the secondary antibodies. Finally, the membrane was exposed using an imaging system, such as the Bio-Rad CHEMIDOC MP (Hercules, CA, USA), to capture the protein bands. The relative protein expression levels were quantified using Bio-Rad Image Lab 3.0 software (Hercules, CA, USA), standardized based on the protein expression levels of GAPDH, and compared to the normalized protein levels of the control cells. The control protein level was set to 1.0 for comparison, and the results represent three independent experiments.

### 4.13. Cell Culture

The 266-6 cell line from mouse pancreatic acinar cells was obtained from the Department of Integrated Traditional Chinese and Western Medicine at West China Hospital and was cultured in DMEM containing 10% fetal bovine serum and 100 μg/mL streptomycin and penicillin in an incubator maintained at 37 °C with 5% CO_2_.

### 4.14. Cell Viability Assay

The CCK-8 assay was performed for cell viability. The cells were treated with different concentrations of NINJ1_26-37_ (15, 20, 25, 30, and 35 μM), RITA (3, 6, 9, 12, and 15 μM), and PFT-α (3, 6, 9, 12, and 15 μM) with STC (1.5 mM) for 24 h. At the endpoint, 10 μL of CCK-8 was added to every well and incubated for 4 h. Biotex Synergy Mx microplate reader (EL Segundo, CA, USA) was used for measurements at 450 nm absorbance.

### 4.15. HCS Real-Time Imaging

Primary acinar cells and 266-6 cells were used with Perkin Elmer CellCarrier-96 Ultra (Waltham, MA, USA). Cell carriers were imaged using a 40× or 10× Air objective on Perkin Elmer Opera Phenix Plus HCS System (Waltham, MA, USA) equipped with an environmental controller and gas mixer to maintain cells at 37 °C and 5% CO_2_. The bright field was imaged every 5 min overnight. Images were processed and videos were generated using Perkin Elmer Harmony^®^ (Waltham, MA, USA). The outcome analysis was processed in Perkin Elmer Harmony^®^ (Waltham, MA, USA) using a custom script with a graphic user interface representing data at the field and plate levels.

### 4.16. Statistical Analysis

The data were calculated as the mean ± SEM. One-way ANOVA was used, followed by Dunnett’s post hoc test. Statistical analysis was presented using GraphPad Prism 8.0 (San Diego, CA, USA). The results were calculated using data from at least three independent experiments. *p* < 0.05 was considered statistically significant.

## Data Availability

This article and Supplementary Material contains all data from this study.

## Supplementary Materials

The following supporting information can be downloaded at https://www.mdpi.com/article/10.3390/ijms241411554/s1.

## Abbreviations

AP	Acute pancreatitis
SAP	Severe acute pancreatitis
PMR	Plasma membrane rupture
DAMPs	Damage-associated molecular pattern
NINJ1	Ninjurin 1
ER	endoplasmic reticulum
IP3R	inositol 1,4,5-trisphosphate receptor
ΔΨm	mitochondrial membrane potential
MCU	mitochondrial calcium uniporter
AML	Amlodipine
STC	sodium taurocholate
HCS	High-content screening
SOD	superoxide dismutase
MDA	malondialdehyde
