# Supplementary material for: Calcium/P53/Ninjurin 1 Signaling Mediates Plasma Membrane Rupture of Acinar Cells in Severe Acute Pancreatitis
Source: Int J Mol Sci. 2023 Jul 17;24(14):11554. doi: 10.3390/ijms241411554 (PMC10380776; doi:10.3390/ijms241411554)
Supplement: Supplementary file 1 [file ijms-24-11554-s001.zip › Supporting Information last.pdf]

Supporting Information

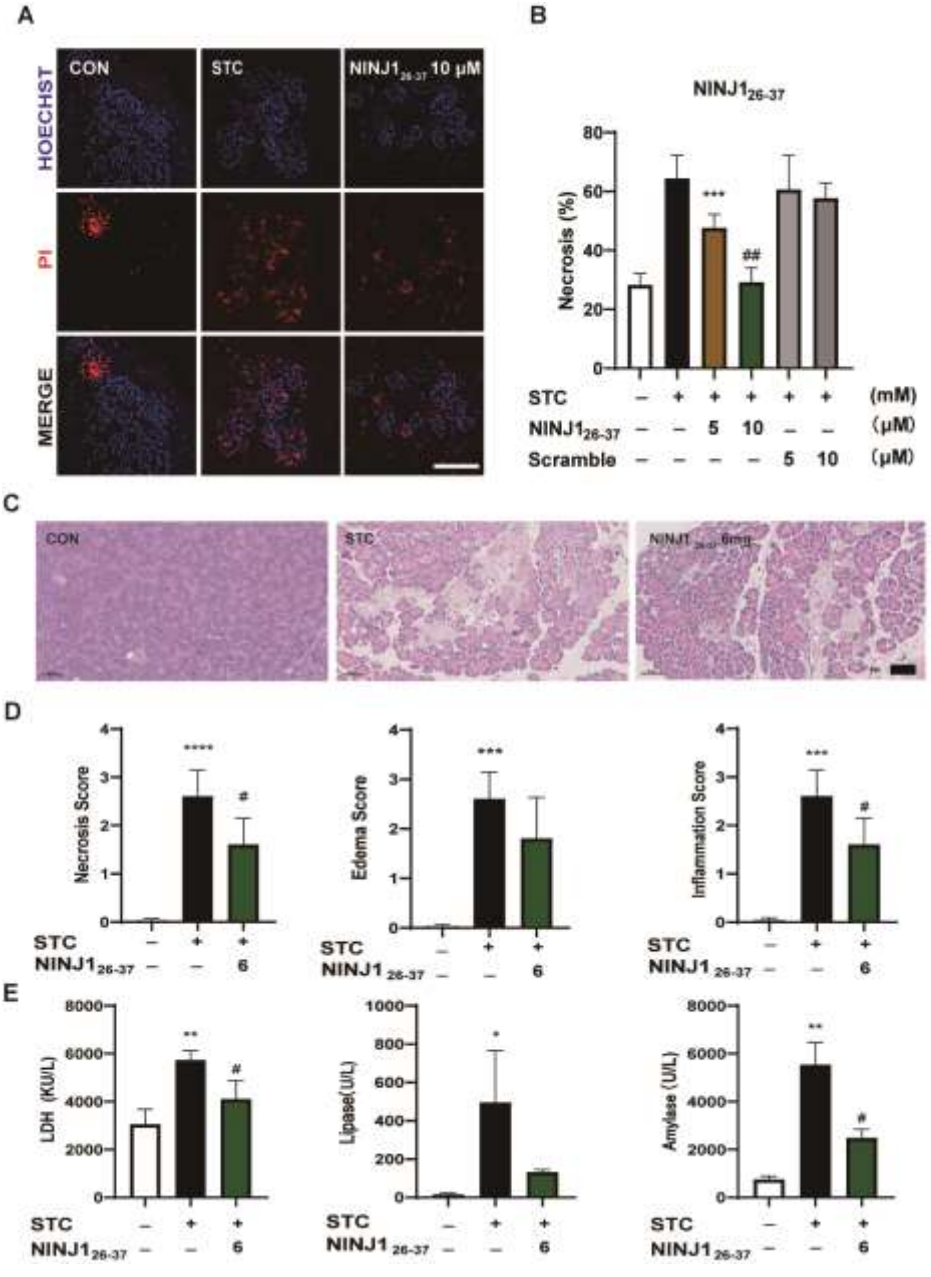

Figure S1 NINJ1$_{26-37}$ effect on the pancreas of STC-SAP mice. (A) Primary mouse acinar cell were incubated with NINJ1$_{26-37}$ (10 μM) and 5mM STC for 50 min; Representative fluorescence plot of PI/Hoechst 33342 staining in primary mouse acinar cell. (B) Column of PI/Hoechst 33342 staining necrosis in primary mouse acinar cell (n = 5). Scale bar = 200 μm. (C) NINJ1$_{26-37}$ was injected intraperitoneally before modeling (3 and 6 mg/kg), once a day, three times in total; SAP was induced by retrograde injection of 3.5% STC into the pancreaticobiliary duct. Samples were taken 24 hs later. Scale bar = 50 μm. (D) the histopathological score of the mouse pancreas (n = 3). (E) Serum levels of lactate dehydrogenase, lipase, and amylase were measured (n = 3). STC: sodium taurocholate;

AML: amlodipine. All data are presented as mean ± SEM, $^{**}P < 0.01$, $^{***}P < 0.001$, $^{****}P < 0.0001$ *vs.* the control group; $^{\#}P < 0.05$, $^{\#\#}P < 0.01$, $^{\#\#\#}P < 0.001$ *vs.* the STC group.

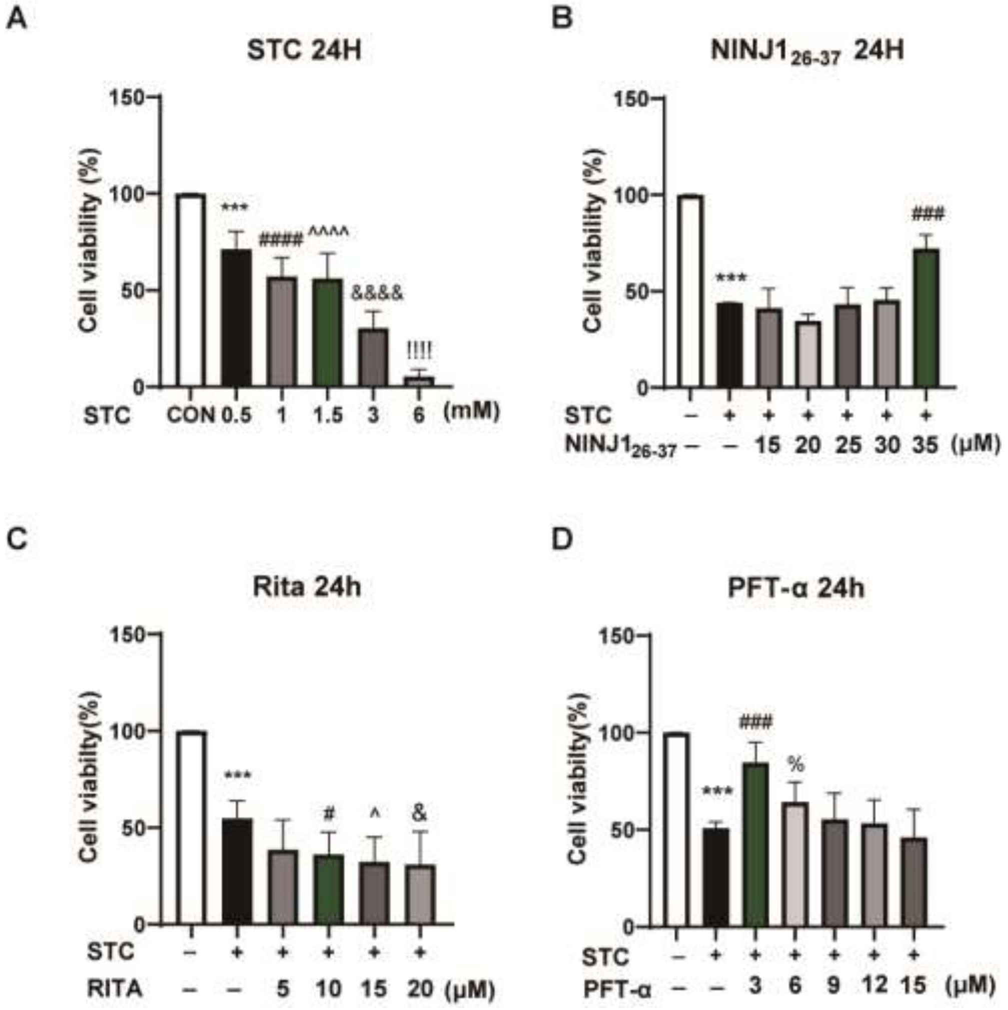

Figure S2 Effect of NINJ1$_{26\text{-}37}$, PFT-α and RITA on cell viability treated with STC. (A) CCK-8 assay detected the viability of 266-6 cells treated with different STC concentrations (0, 0.5, 1, 1, 3, 6 and 0.5μM) for 24 hs. (B)After 266-6 cell were treated with different concentrations of NINJ1$_{26\text{-}37}$ (15, 20, 25, 30 and 35 μM) and STC (1.5 mM) or 24 hs, the cell viability was detected by CCK-8 assay. (C) After 266-6 cell were treated with different concentrations of PFT-α (3, 6, 9, 12 and 15μM) and STC (1.5 mM) for 24 hs, the cell viability was detected by CCK-8 assay. (D) After 266-6 cell were treated with different concentrations of RITA (3, 6, 9, 12 and 15μM) RITA and STC (1.65mM) for 24 hs, CCK-8 assay was used to detect the cell viability. STC: sodium taurocholate; RITA: P53 agonist; PFT-α: P53 inhibitor. All data are presented as mean ± SD; n = 5; $^{***}P < 0.001$ *vs.* the control group, $^{\#}P < 0.05$, $^{\#\#\#}P < 0.001$, $^{\#\#\#\#}P < 0.0001$, $^{\wedge}P < 0.05$ $^{\wedge\wedge\wedge\wedge}P < 0.000$, $^{\&}P < 0.05$, $^{\&\&\&\&}P < 0.0001$, $^{!!!!}P < 0.001$ *vs.* the STC group.

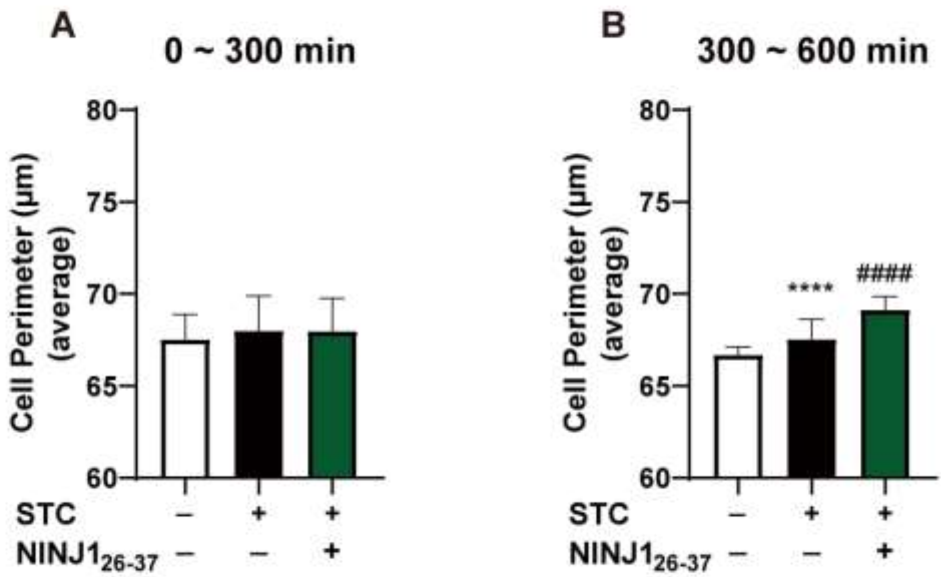

Figure S3 Effect of NINJ1$_{26\text{-}37}$ on membrane perimeter of 266-6 acinar cell (A) Calculate the average cell circumference during the time period of 0-300 min. (B) Calculate the average cell circumference during the time period of 300-600 min. STC: sodium tauroursodeoxycholate. NINJ1$_{26\text{-}37}$: NINJ1-blocking peptide. All data are presented as mean ± SEM, $^{****}P < 0.0001$ *vs.* CON group. $^{\#\#\#\#}P < 0.0001$ *vs.* STC group.

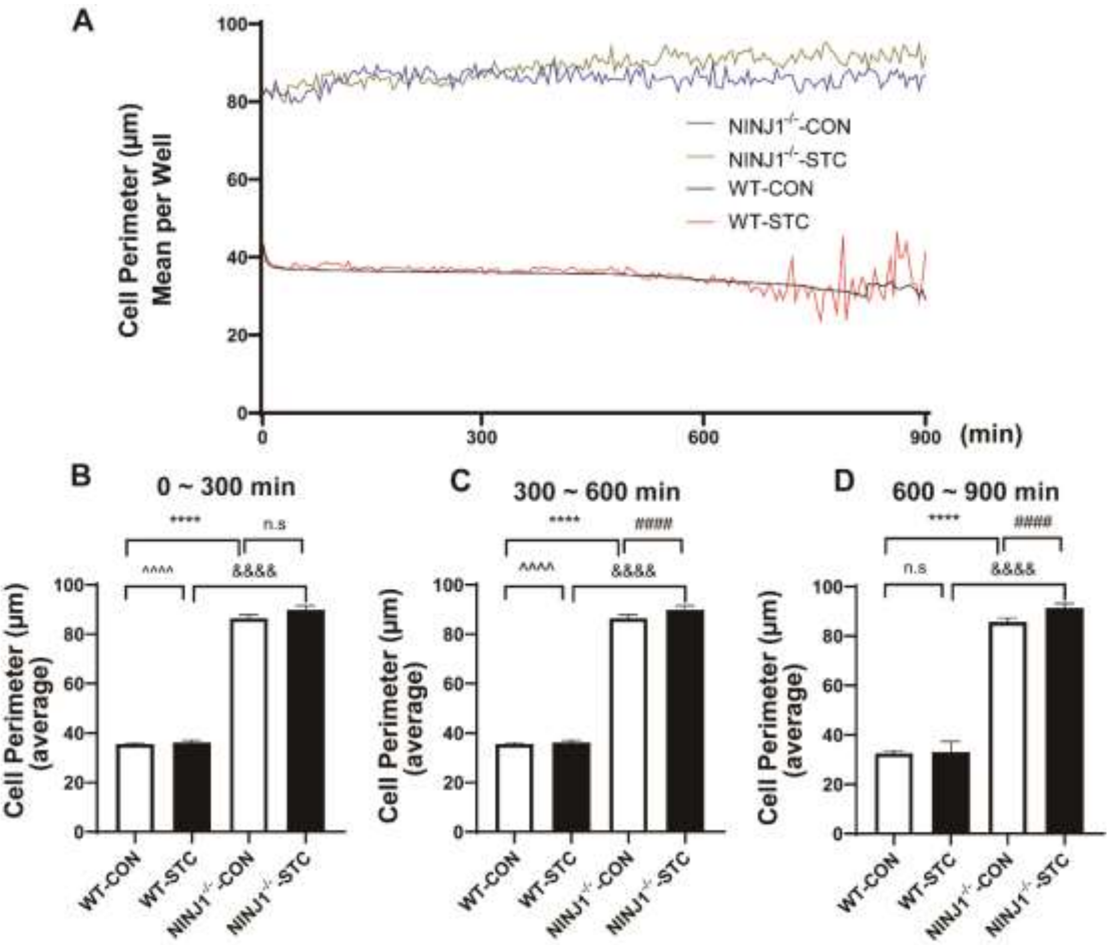

Figure. S4 Effect of NINJ1 $^{-/-}$ primary mouse pancreatic acinar cell membrane perimeter. (A) HCS data analysis system was used to analyze the changes in cell perimeter at different time points. STC: sodium taurocholate (n = 3). (B) Statistical cell mean perimeter in the 0-300 min time period. (C) Statistical cell mean perimeter in the 300-600 min time period. (D) Statistical cell mean perimeter in 600-900 min time period. STC: sodium taurocholate. NINJ1$^{-/-}$: NINJ1 knockout mice. All data are presented as mean ± SEM, $^{****}P < 0.0001$ WT-CON *vs.* NINJ1$^{-/-}$-CON, $^{####}P <$ 0.0001 NINJ1$^{-/-}$-CON *vs.* NINJ1$^{-/-}$-STC, $^{\wedge\wedge\wedge\wedge}P < 0.0001$ WT-CON *vs.* WT-STC, $^{\&\&\&\&}P <$ 0.0001 WT-STC *vs.* NINJ1$^{-/-}$-STC.

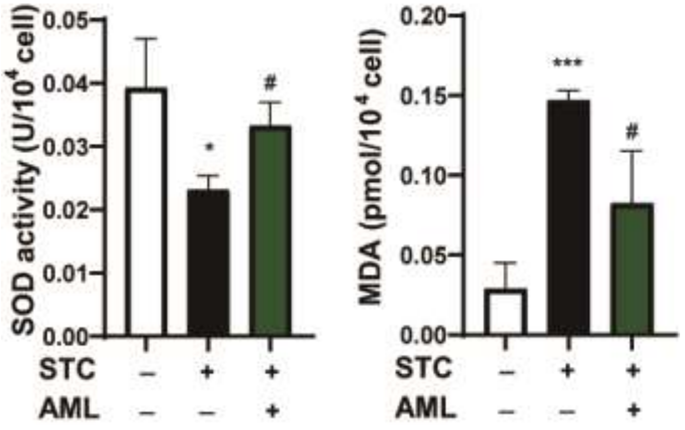

Figure. S5 Effect of AML on serum oxidative stress levels in STC-SAP mice. Detection of SOD and MDA in the blood supernatant. All data were expressed as mean ± SEM, n = 3; * $P < 0.05$, ***P < 0.001 *vs.* the control group. $^{\#}$ $P < 0.05$ *vs.* the STC group.

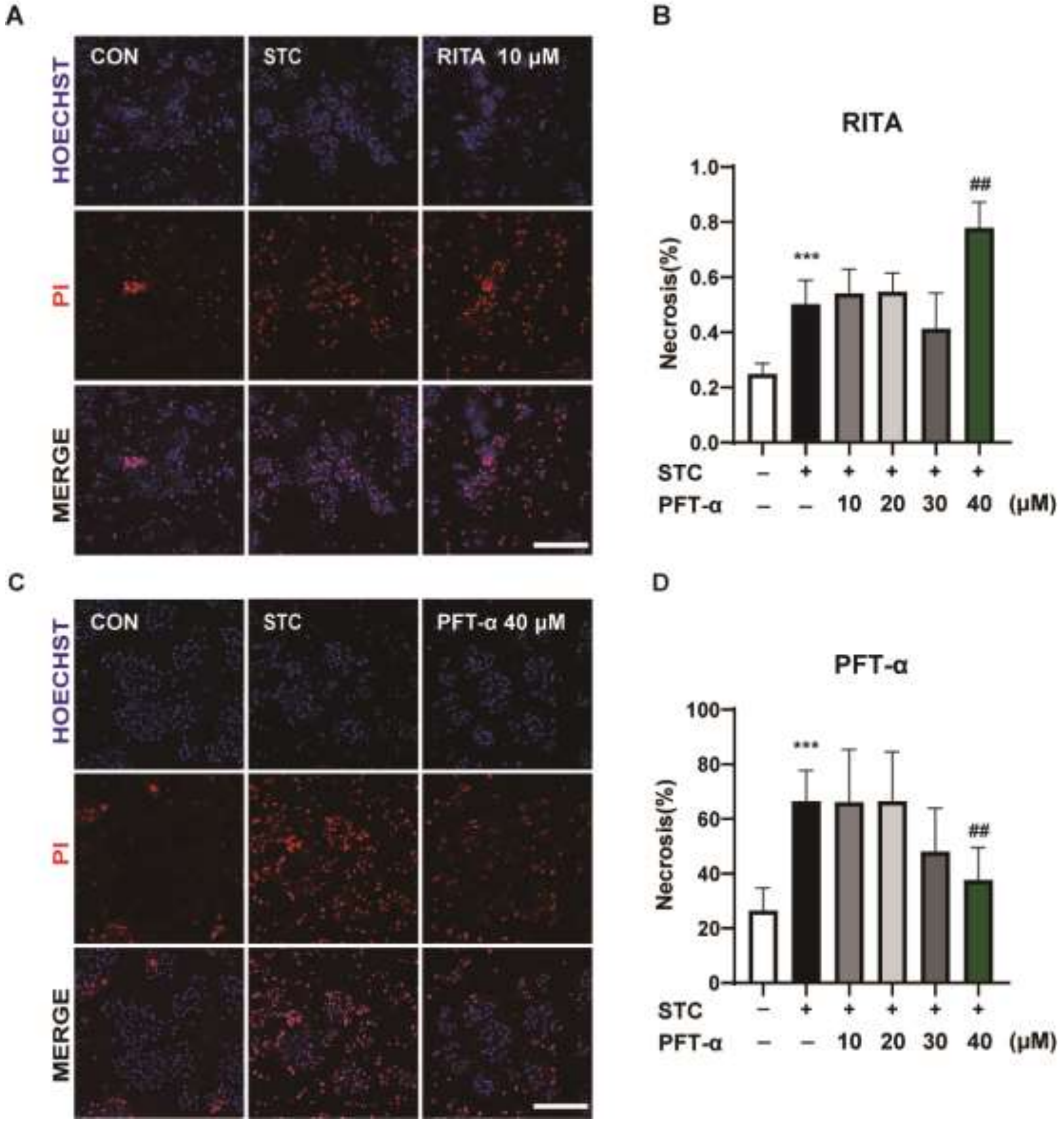

Figure S6 Effect of PFT-α and RITA on primary mouse acinar cell necrosis. Different concentrations of PFT-α (10, 20, 30 and 40 μM) or RITA (0.1, 1, 5 and 10 μM) with STC (5 mM) were incubated for 50 min. (A) Representative fluorescence plot of Hoechst 33342/PI staining in primary mouse acinar cell incubating by RITA. (B) Column diagram of Hoechst 33342/PI staining necrosis in primary mouse acinar cell incubating by RITA. (C) Representative fluorescence plot of PI/Hoechst 33342 staining in primary mouse acinar cell incubating by PFT-α (D) Column diagram of PI/Hoechst 33342 staining necrosis in primary mouse acinar cell incubating by PFT-α. STC: sodium taurocholate; AML: amlodipine. RITA: P53 agonist; PFT-α: P53 inhibitor. All data are

presented as mean ± SEM, n = 5; $^{****}P < 0.001$, **** $P < 0.0001$ *vs.* the control group; $^{\#\#}P < 0.01$, $^{\#\#\#\#}P < 0.0001$ *vs.* the STC group. Scale bar = 200 μm.

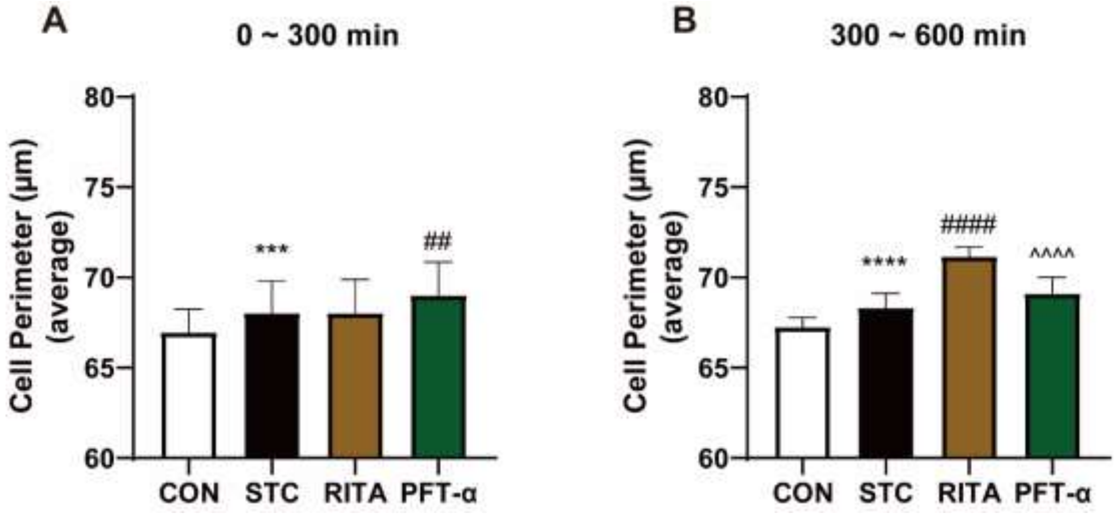

Figure. S7 Effect of RITA and PFT-α on membrane perimeter of 266-6 acinar cell. (A) HCS data analysis system was used to analyze the changes in cell perimeter at different time points. STC: sodium taurocholate（n = 3）. (B) Statistical cell mean perimeter in the 0-300 min time period. (C) Statistical cell mean perimeter in the 300-600 min time period. STC: Sodium taurocholate. AML: amlodipine. All data are presented as mean ± SEM, $^{***}P < 0.001$, $^{****}P < 0.0001$ *vs.* the CON group. $^{\#\#}P < 0.01$, $^{\#\#\#\#}P < 0.0001$ *vs.* the STC group.
